# Reanalysis of historic elemental speciation filters to investigate the presence of fibrous mineral particles using microscopy techniques

**DOI:** 10.3389/fchem.2022.1032624

**Published:** 2022-11-03

**Authors:** Nick Talbot, Kim N. Dirks, Wendy Fan, Hamesh Patel, Seosamh B. Costello, Martin Brook, Perry Davy

**Affiliations:** ^1^ School of Environment The University of Auckland, Auckland, New Zealand; ^2^ Faculty of Engineering The University of Auckland, Auckland, New Zealand; ^3^ Mote Ltd., Auckland, New Zealand; ^4^ GNS Science, Wellington, New Zealand

**Keywords:** erionite, scanning electron microscopy (SEM), reanalysis, PTFE filters, energy dispersive analysis (EDS), archiving

## Abstract

A case is presented for the value of archiving air quality filters to allow for retrospective analysis of emerging contaminants, that is filter constituents not considered to be harmful (and thus not identified or quantified specifically) at the time of collection but subsequently considered to be of interest. As an example, filters from a 20-year historical archive consisting of 16,000 filters from three sites across Auckland are re-examined for the presence of elongated mineral fibres known to be present in rock across the city. Originally collected for the purpose of the source apportionment of particulate matter, 10 filters from each of the three sites were chosen for reanalysis based on their high silica and aluminium content, and thus considered more likely to contain fibre-like particles (FLP). These filters were analysed using various microscopic methods, including phase contrast microscopy (PCM), scanning electron microscopy (SEM) and energy-dispersive x-ray spectroscopy (EDS). The results show that although the commonly used fibrous polytetrafluoroethylene (PTFE) material of the filters may hamper the visual identification of any fibre-like particles under a certain length, their key components are able to be identified using a combination of PCM and SEM when they are of a suitable dimension and have settled in a certain orientation on the filter. In this case, the use of EDS confirmed the silicon content of the fibres and also revealed elemental spectra. Although the exact identification of the mineral fibre is uncertain, the EDS scan is consistent with hazardous zeolites such as erionite, known to be present in the rock found in Auckland. This study highlights the value in maintaining filter archives for the purpose of investigating the historical evolution of emerging atmospheric pollutants.

## Introduction

A challenge associated with assessing the risk posed by emerging atmospheric pollutants is often the lack of historical data available for identifying and quantifying such pollutants so that trends in concentrations can be determined retrospectively. While opportunities do not exist for gaseous pollutants, archives of air filters could provide the records needed in the case of particulate matter, as long as the selected filter material is suitable for the analytical methodologies proposed. Thus, as emerging atmospheric pollutants are identified, historical filters could be data-mined to assess whether the specific pollutant of interest was present in the ambient air, and, if so, in what quantity, as well as how it varied temporally.

New Zealand’s Institute of Geological and Nuclear Sciences (www.gns.cri.nz) maintains a carefully logged, dated, and stored filter archive of air particulate matter samples from across New Zealand (National Air Particulate Speciation Database DOI: https://doi.org/10.21420/R58Q-FZ78). The database dates back over 20 years and consists of over 40,000 samples, including a long historical record captured at various sampling locations across the Auckland region (Talbot and Lehn, 2018). The filters that form the archive were originally sampled and analysed using non-destructive techniques (Ion Beam Analysis or X-ray fluorescence spectroscopy) to extract elemental compositional data for use in receptor modelling and apportionment of the sources of urban air particulate matter pollution (Davy et al., 2012; Davy et al., 2017a). The filters, primarily 47 mm diameter polytetrafluoroethylene (PTFE) or polycarbonate membrane filters, have since been stored individually in sealed petri dishes located in cabinets held at room temperature. Thus, the filters remain in-tact and suitable for reuse.

Over recent years there has been increased awareness that fibre-like particles might be present in the air across Auckland. This has come to attention due to work regarding silicas and, more recently, fibrous zeolites, with specific attention paid to erionite. Erionite is a Category 1 carcinogen (IARC, 1983), with exposure linked to malignant mesothelioma and other lung cancers in regions where it has been found in disturbed bedrock (Dogan et al., 2006; Carbone et al., 2011; Gualtieri et al., 2016) and where it has been found to be present on the near-surface dust and soil (Van Gosen et al., 2013; Harper et al., 2017; Beaucham et al., 2015).

**FIGURE 1:**
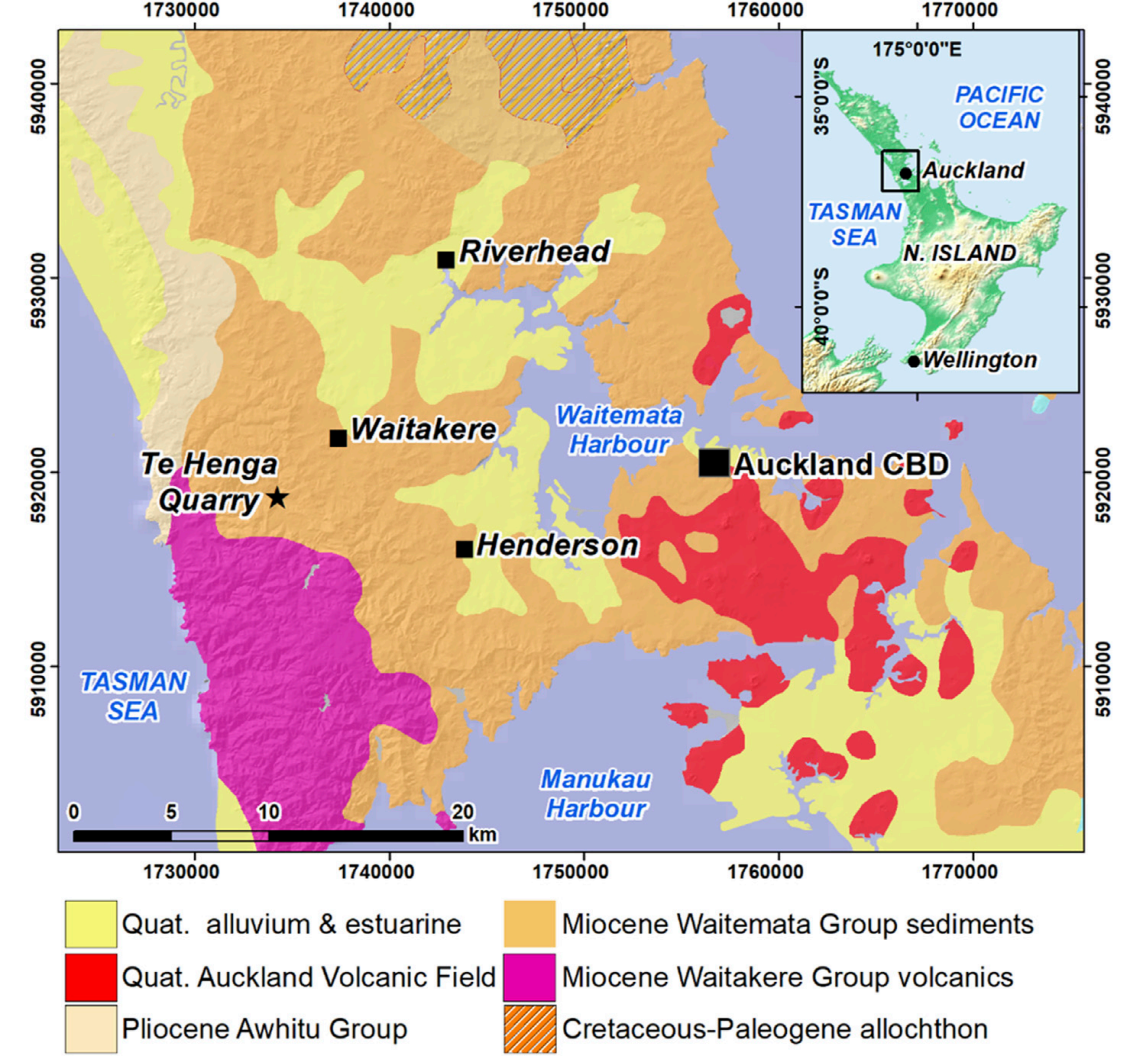
Generalised geology of the Auckland region, including sites where erionite has been identified, as well as the location of the Henderson historical dataset.

Zeolites, including fibrous erionite, have been identified in several locations in the Miocene Waitemata Group sediments (Sameshima 1978), exposed across much of the Auckland urban and suburban regions. For example, in the upper Waitemata Harbour at Riverhead (Davidson and Black, 1994; Patel and Brook, 2021), fibrous erionite has been identified in riverbank exposures underneath a thin veneer of Quaternary sediments, as well as in rock outcrops in shore platforms in the intertidal zone. Erionite has also been identified in the Miocene Waitakere Group volcanics at Te Henga Quarry −9 km to the west of Henderson, in the west of Auckland (Brook et al., 2022). The quarry was operational until 2015 and used for aggregate minerals, as well as in a cutting at the nearby Waitakere Railway Station (Figure 1).

Thus, erionite, along with other fibrous silicas and zeolites, can be considered to be emerging contaminants in Auckland. Their importance in this specific context is especially relevant given that the risk of their disturbance has increased in recent years with Auckland’s rapid urban expansion across previous greenfield sites (Stats NZ, 2021). Moreover, there has been increased awareness recently about the risk posed by fibrous mineral forms including silicas (Otsuki et al., 2016; Sanchez et al., 2009) and zeolite minerals such as erionite (Brook et al., 2020; Patel and Brook, 2021). In this study, the GNS filter archive is used to investigate its potential to reconstruct the historical evolution of fibrous silicas and zeolites in the air across Auckland.

An important limitation is that there is currently limited literature on methods for identifying and quantification of erionite on filters collected from air sampling (Beaucham et al., 2015; Carbone et al., 2011), with no apparent standard methods followed. However, the literature suggests that filter measurement techniques encompassing microscopic analytical methodologies similar to those used for asbestos quantification (such as those provided by NIOSH 7400 and 7402 and ISO 13002) are potentially the most suitable (NIOSH et al., 1999). The instruments proposed include the scanning electron microscopy (SEM), an instrument often used in the analysis of air particulate matter (González et al., 2018; Li and Shao, 2009). SEM techniques have been used in numerous studies in both quantitative and qualitative analysis of filter samples, including in the analysis of particle size, morphology and composition to infer sources (Triplett et al., 2010). In addition, when combined with energy-dispersive x-ray spectroscopy (EDS), this method provides information about both the morphology and the chemical composition of the particles present on the filter (Dogan, 2003).

This study investigates whether a historical archive of air filters, using standard PTFE or polycarbonate membrane filters, can be used to identify and quantify the presence of an emerging contaminant, in this case erionite, using a combination of phase contrast microscopy (PCM) and SEM, coupled with EDS microscopic techniques. The result of the study highlights the value of retaining historical archives for the purpose of retrospective air pollution analysis.

## Site selection

**FIGURE 2:**
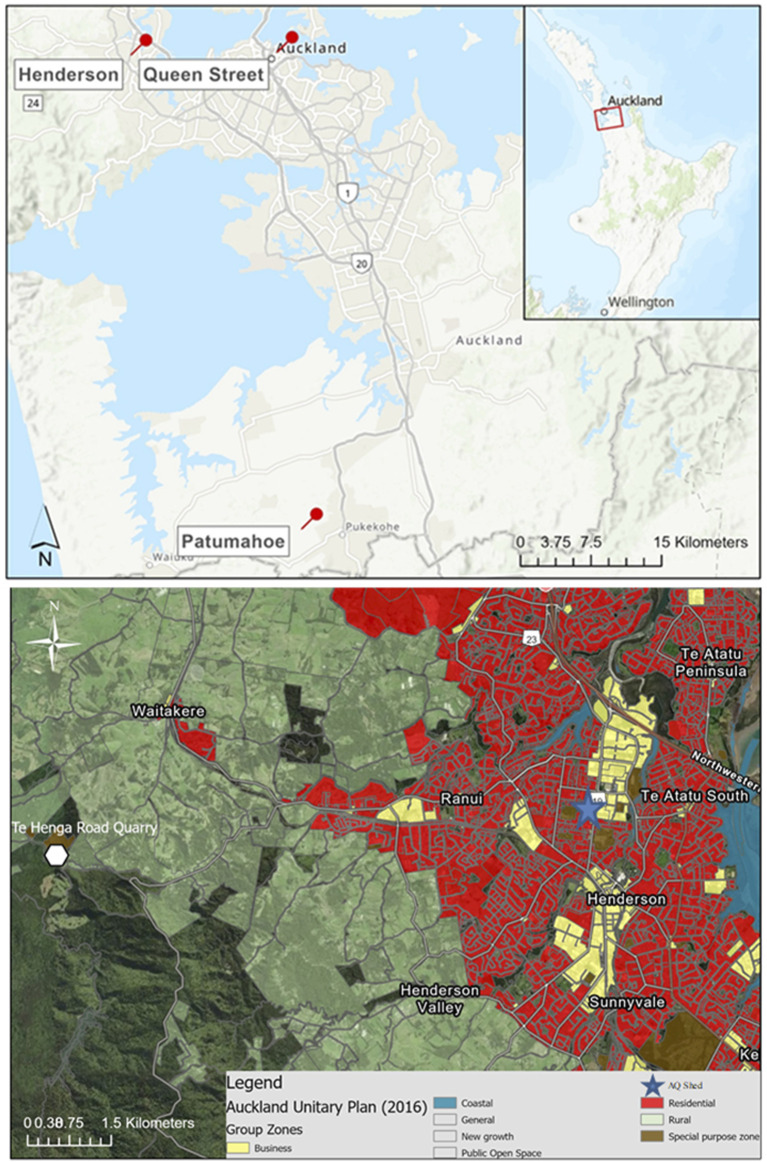
Map of the three sites where the elemental analysis of filters was carried out (top), and a GIS land use map of the Henderson site (below), Henderson being the key site of interest for this work.

For the initial screening process, three sampling sites were chosen to represent areas typically affected by substantially different airborne pollutants. These locations were Patamahoe (rural countryside location away from dwellings and busy roads), Queen Street (a central Auckland main shopping street), and Henderson (a residential/light industrial suburb in the West of Auckland, with sampling carried out at a site set alongside a major arterial road) (Figure 2). The Henderson site was established in 1997 as part of Auckland’s air quality monitoring network and is of particular interest, as it is located within 5 km of known locations of erionite-bearing rock strata (Patel et al., 2021).

## Filter selection process using source apportionment data

Filter-based particulate matter samples were collected at each of the chosen sites on a 24-h time-integrated sampling basis using reference monitoring methods. Sampling for chemical speciation analysis began at Queen Street and Henderson from 2006 onwards, and for Patamahoe, sampling was conducted during 2010 only.

Particulate matter elemental compositional analysis [Na to U by accelerator-based Ion Beam Analysis (Barry et al., 2012)] and source apportionment data (receptor modelling by positive matrix factorization (Davy et al., 2017a; Sun et al., 2020) were used as an initial tool to identify the most suitable filters for reanalysis. The mass content of each element upon each filter was obtained from the historical data archives (National Air Particulate Speciation Database DOI: https://doi.org/10.21420/R58Q-FZ78), and these data were used to establish a subset of filters from each site with the highest soil/mineral content.

**FIGURE 3:**
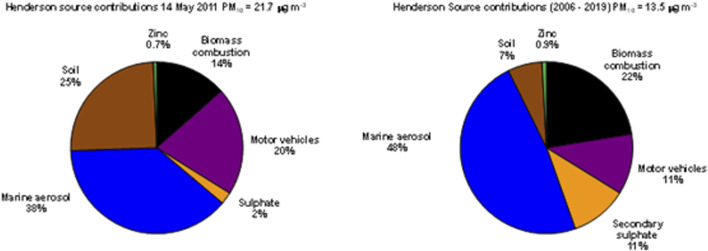
Source apportionment from chemical analysis taken from the filter on 14 May 2011 (left) compared to the long-term dataset from the Henderson site (right**)**.

The criteria used were as follows; filters with high Al and Si content were most likely to contain substantial quantities of crustal matter particles since crustal matter is largely dominated by aluminosilicate minerals (Lide 1992). While any ambient air particulate sample could possibly contain fibrous mineral particles, only filters with high levels of aluminium (Al) and silica (Si) mass from compositional analysis and a corresponding crustal matter source component as evidenced by the source type (Soil for the Henderson and Patumahoe sites, Soil and Construction dusts for the Queen Street site) and corresponding source chemical profile (Supplementary Figures S2; Supplementary Table S1) were considered for further investigation to maximize the potential for identifying fibrous mineral entities. Due to the labour-intensive nature of filter screening, a total of 30 filters, 10 from each of the three chosen sites, were selected for further analysis based on the above criteria. Figure 3 is an example of source apportionment results for the filter from the Henderson site from 14 May 2011, a filter high in soil content based on the composition of Al and Si components (23% compared to the long-term average of 7%).

## Analysis of the filters using microscopy techniques

In the first instance, phase contrast microscopy was undertaken on all 30 of the filters, 10 from each of the three air quality sites. Two different microscopes were used to examine the filters, each offering a slightly different magnification quality: A Leica DM2700P Petrographic Microscope and a Leica MZ16 Binocular Microscope. In each case, the filters were scanned in their entirety to visually assess the presence and density of fibrous material cover. A careful record of the morphology (shape and visual dimensions) was made of the deposited material on each filter so the data could be referenced for later analyses. Both microscopes were network linked and enabled with digital imagery capabilities. Once fibrous materials had been located using the microscopes, the filters were set aside ready for SEM-EDS analysis.

A crucial part of this work is to test whether the fibrous nature of the PTFE filter would allow for identification of fibres on top of, or within, the filter itself. It is clear that most fibres are quite clearly identified as they are both larger than the fibres of the filter, and also generally sit at angles that are not consistent with the filter. An attempt to measure the typical woven fibre within the filter was made, however, the fibres were not consistent so no representative value could be obtained. However, it was noted that almost all woven fibres within the filter were less than 1 µm in length. It is, therefore, reasonable to assume that any fibres of interest less than 1 µm in length could be difficult to differentiate from the filter medium.

### Phase contrast microscopy (PCM)

The PTFE filters are dense with a tightly woven fibrous medium which restricted the amount of light able to penetrate through the filter, as shown in Supplementary Figure S1. Even a blank (clean) filter presented quite a dull image. To try and improve this, an external table lamp was used to increase luminosity. However, this approach had limited effectiveness, as shown in Supplementary Figure S1.

The dullest filters were found to be those from the Queen Street site, due in part to this site having the largest loading of combustion particles, such as black carbon, which absorbs light effectively (Supplementary Figure S1B). Overall, however, very few fibres were found in any of the ten filters chosen from the Queen Street site. Similarly, for Patamahoe, a rural background site set in farmland, the filters often contained complex structures that morphology seemed to suggest were of biological origin (Supplementary Figure S1D). The filters with the most visible fibre-like participles (FLPs) were those from Henderson, with FLP found in several of the filters around the same period in 2011 (Supplementary Figure S1C).

**FIGURE 4:**
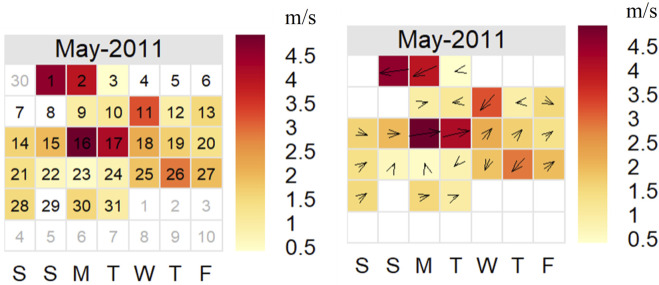
Calendar plot showing wind speed and direction by day for May 2011. The darker colours indicate higher winds, the arrows the prevailing direction for that day. The key provides wind speed in units of m/s with the days of the week along the x-axis.

After careful investigation using PCM, the filter with the largest concentration of fibres was selected and became the focus of attention. That filter (24-h time integrated sampling, 16.7 L per minute sampling rate, 23 m^3^ total volume of air sampled), from the Henderson air quality site, was collected on 14 May 2011. This filter was chosen due to visual evidence of fibres using the above PCM and source apportionment data, with the highest fibre count over 30 planes of vision (Supplementary Table S2). The 30 planes of vision covered a small fraction of the overall effective filtration surface area of 1,195 mm^2^. The methodology was not designed to offer a quantification of fibres on filters, rather whether fibres could be located and identified using this methodology. The full dataset of filters for May 2011 were then analysed with SEM/EDS techniques (a total of 8 filters, one sample every third day) on the basis that it could be expected that the disturbance in mineral fibres leading to the presence of fibres on the filter of 14 May 2011 would likely have contaminated other filters collected around that time (say over a period of a month). Also, the Henderson site sits downwind of a site of active disturbance of known erionite, the Te Henga quarry. As shown in Figure 4, westerly winds prevailed across Auckland city during May 2011, thus the prevailing wind was favourable for possible contamination.

### Scanning electron microscopy with electron differential X-ray spectroscopy

The pre-processing and processing method for SEM-EDS use were as follows. Filter samples for analysis were prepared by cutting a small square (5 × 5 mm) from each Teflon filter and attaching it to a 12.5 mm aluminium stub with carbon glue. Each sample was then sputter-coated with 10 nm Pt using a JEOL Smart Coater 290308CRT. The coated filters were then degassed using a PD3 Edwards Plate Degasser. The coating process is necessary to prevent sample charging (due to the electron beam) during analysis which affects imaging and resolution. Once prepared, filter samples were inserted into the electron microscope for analysis. The filter samples were then placed in a multi-stub holder, with SEM-EDS analysis conducted using a JEOL JCM-6000 with an accelerating voltage of 15 KV.

**FIGURE 5:**
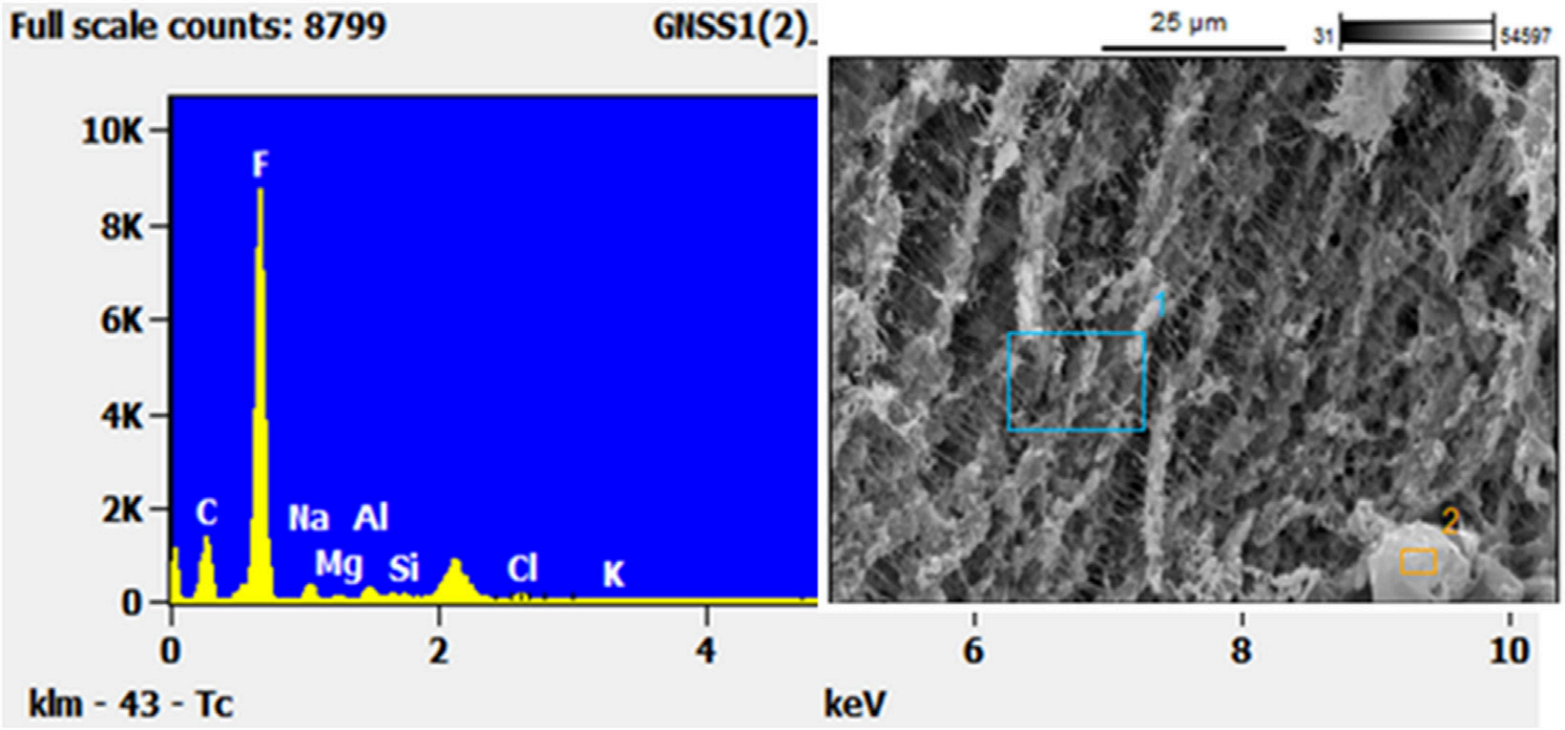
SEM/EDS analysis of a blank PTFE filter to show the reader the “background” composition of such filters. Note the very high concentration of Fluorine.

An example image of a blank PTFE filter, shown under SEM, is presented in Figure 5. The fibrous PTFE material is evident under SEM, with intense (high x-ray counts) fluorine peaks identifiable in the EDS spectra. These components are important to understand so as not to confuse particles that look foreign on the filter with the filter itself.

SEM is useful for determining aerosol particle size distributions, particle morphology and particle composition to assist with source identification (Dogan, 2003). EDS is usually coupled with electron microscopic techniques to help identify the chemical components of materials under investigation. In terms of limitations, SEM analysis assumes a flat surface. In addition, larger, coarse mode particles and fibres can block and intercept X-rays producing shadow areas (Moreno et al., 2003). Finally, the high voltage electron beam can penetrate through the particle resulting in partial analysis of underlying particles or filter substrate, whilst potentially damaging the investigated fibre (Ray, 2020).

**FIGURE 6:**
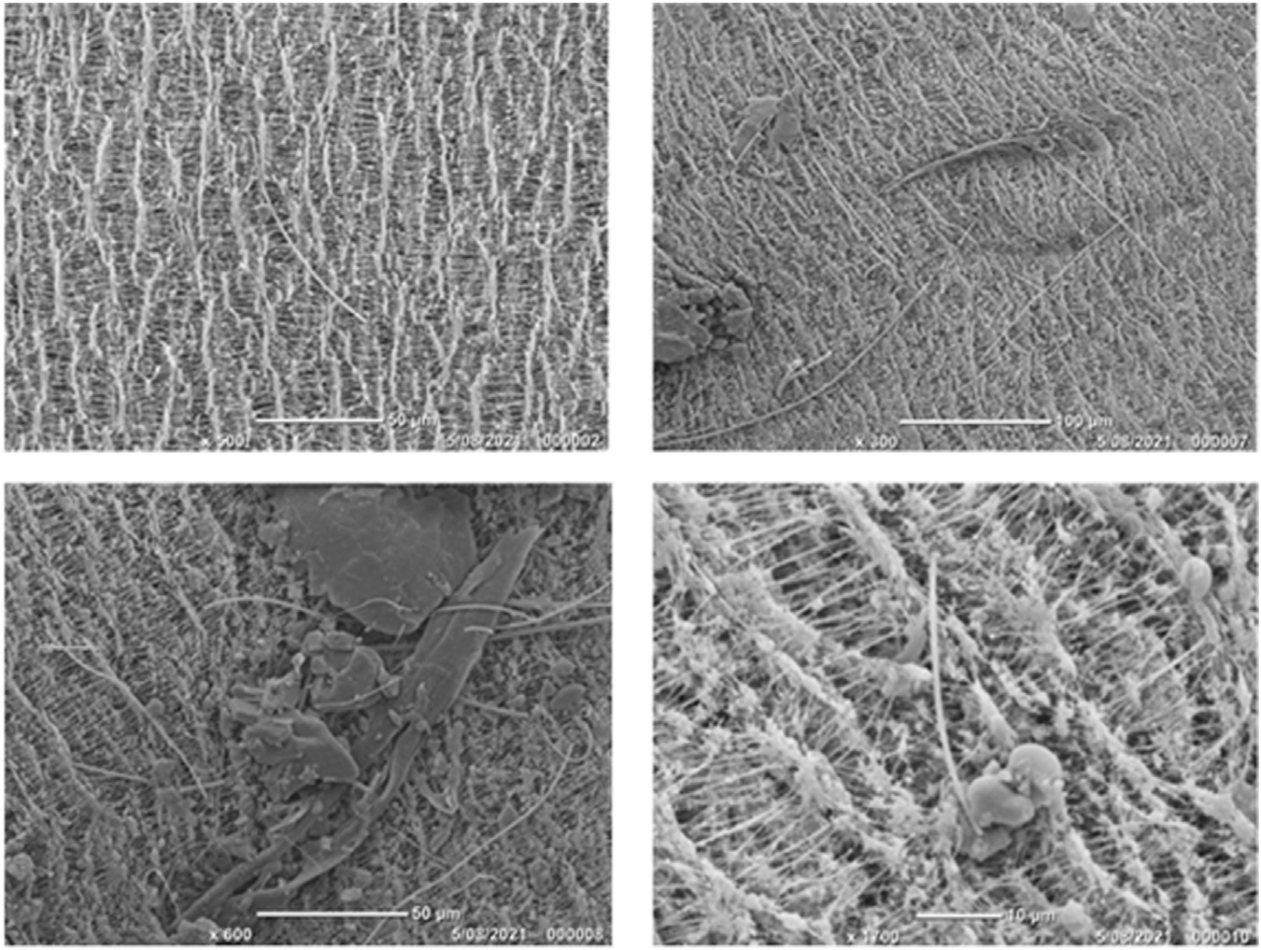
SEM photographs of Henderson filters from May 2011 showing different fibrous materials embedded upon and within the PTFE filters. The images show that although the filter medium is fibrous, fibres can still be identified sitting upon the filter.

Figure 6 shows an example of fibrous materials on a filter collected in Henderson. The scaler at the bottom of each image shows that, although the fibres are long, they are very thin and needle-like in morphology. The SEM photos also highlight the fibrous nature of the filters with a woven texture that could make smaller fibres more difficult to identify, especially if the fibres are embedded in the filter themselves. However, it is evident from Figure 6 that the needle-like FLPs collected from ambient air in Henderson are more easily discernible from filter fibres using SEM than with PCM. However, the cost and time taken to prepare and analyse filters using SEM suggest that there is benefit in using PCM in the first stage of investigation.

**FIGURE 7:**
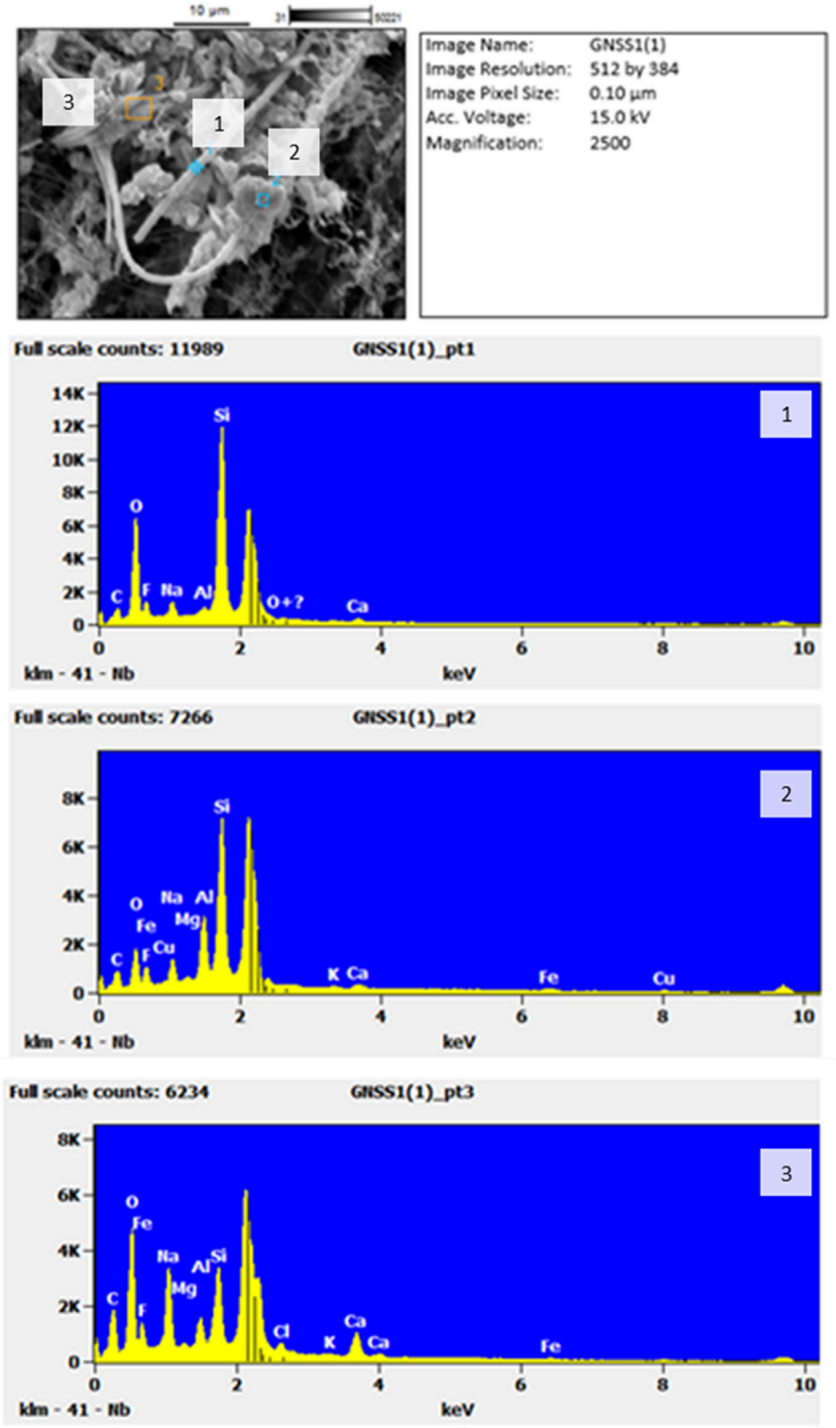
SEM of fibres and particles found on the Henderson filter on 14^th^ May 2011 (top) and EDS scans (below) showing (1) the long straight fibre, (2) crustal fragment and (3) sea salt crystals. The unmarked peak is platinum (Pt), used as splutter coating for the EDS scans.

The SEM image in Figure 7 shows a complex array of different particle structures captured on the filter. A long thin fibre is again clearly discernible from the filter, whilst, alongside it, there are numerous other particles with different morphological structures. Chemical analysis with EDS shows that Si-rich fibres are present. They appear to be long, thin, and respirable. Along the fibres, there are sea salt crystals that appear to have impacted on the fibre, most likely during the sampling process, as well as soil particles of different shapes sizes and of different chemical make-up. However, Al, which is also elevated in the EDS scans shown in Figures 7B,C is a good indicator of aluminosilicate crustal matter.

**FIGURE 8:**
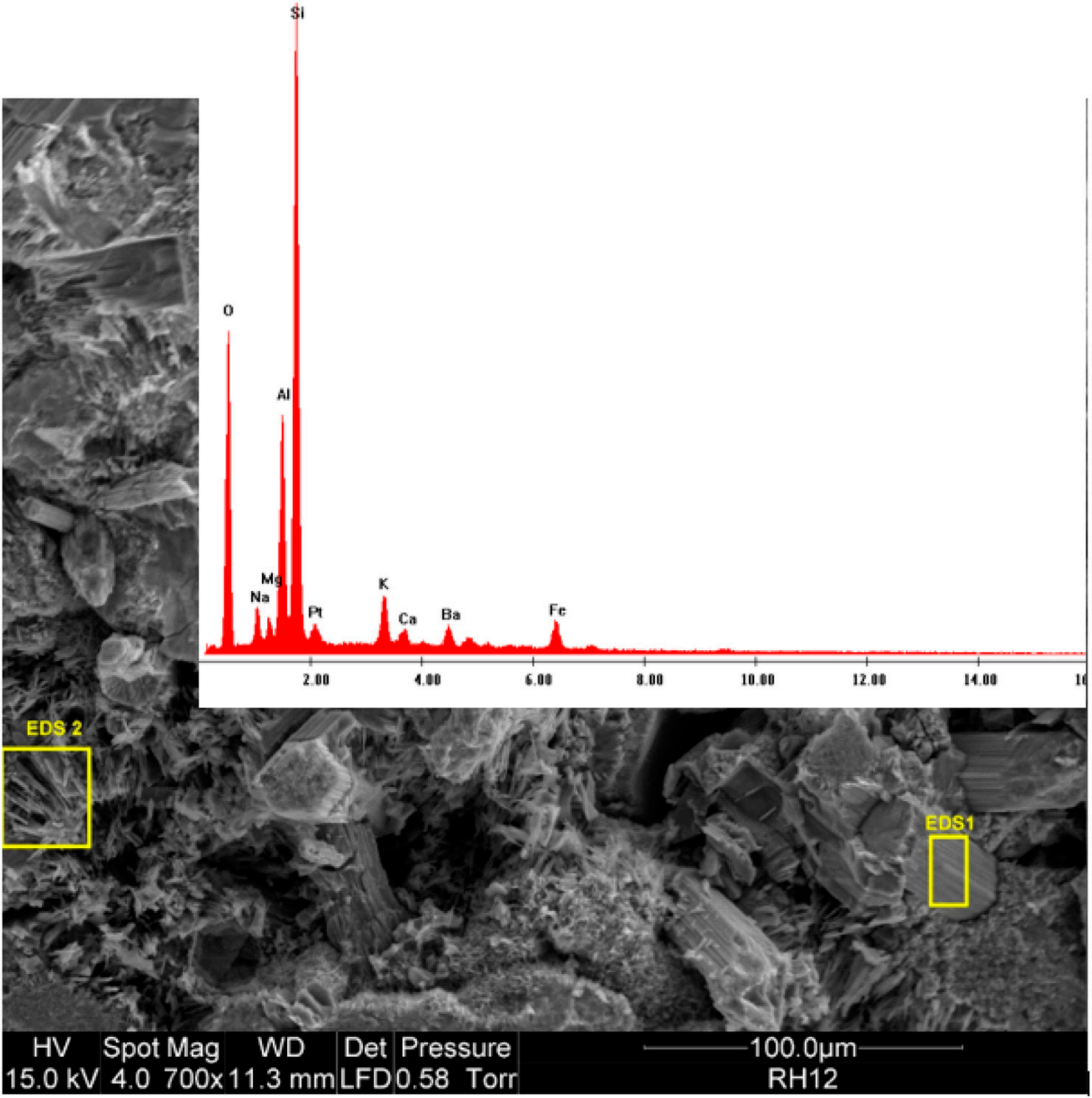
SEM image with EDS relative abundance spectra of known erionite fibres found in Riverhead (Patel et al., 2021).

EDS spectra Figure 7 for the crustal fragment 2) closely matches that of erionite. To compare the results from the Henderson filter, Figure 8 shows a recent SEM with relative intensity EDS scan taken from a bedrock sample in the township of Riverhead, West Auckland, and known to be erionite. Note that with all of the EDS scans, there is an unmarked peak at around 2.1 Kv: this is the platinum coating used as a conductive coating during the reanalysis process.

**FIGURE 9:**
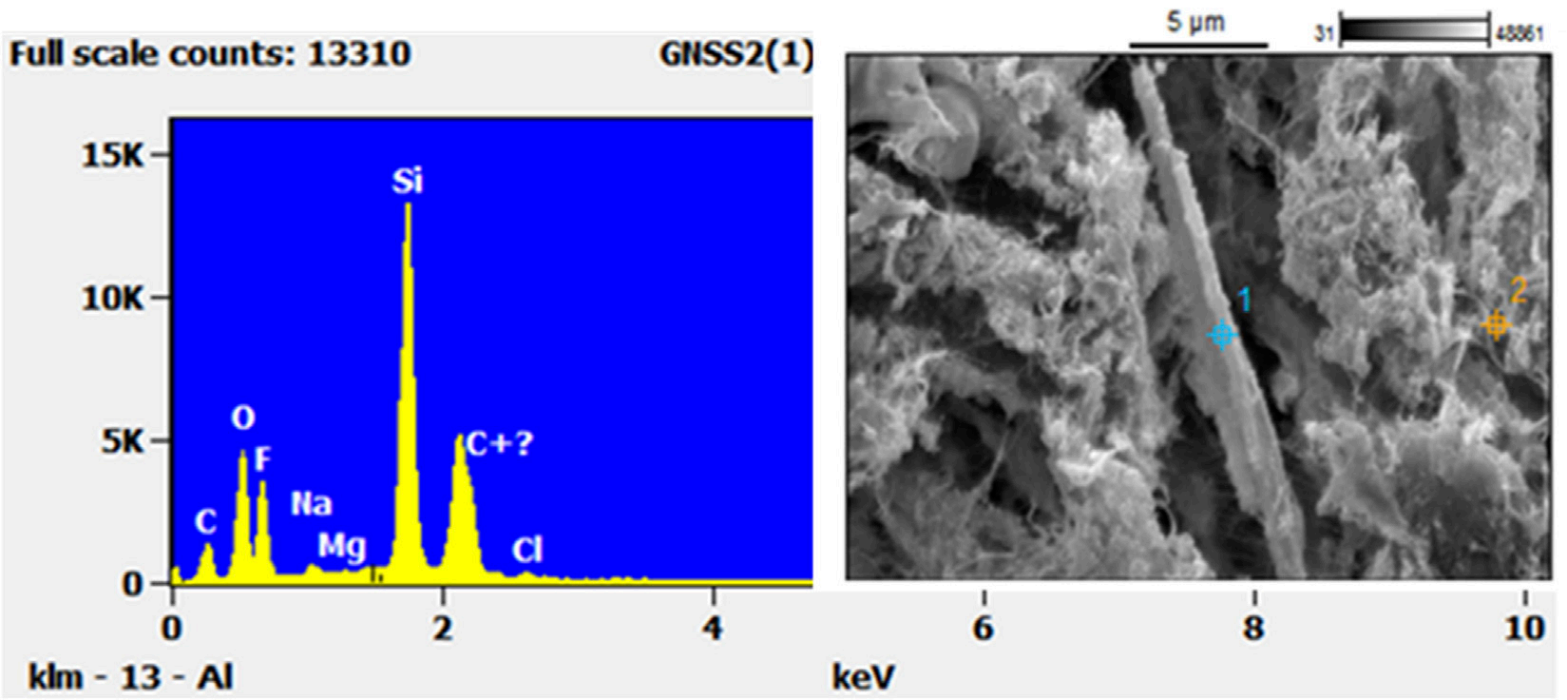
SEM-EDS of mineral-looking fibre found on the Henderson filter from 14 May 2011.

Not all of the high Si fibres look the same. There are also Si-rich fibres that appear to be more like hexagonal clustered structures than elongated in nature. Figure 9 shows such a cluster embedded within the fibres of the filter. These shards are smaller than the fibres shown in the previous images and are morphologically unique. The EDS scan, however, shows that the chemical composition of this fibre is very similar to that shown in Figure 8.

## Discussion

In this study, a historical archive of filter data, originally collected for the purpose of compositional analysis for receptor modelling, was used to investigate the presence of FLP in filters. The elemental composition of particulate matter collected on filters was used to identify the main chemical fingerprint of crustal matter, and a sample of filters with elevated quantities of Si and Al were investigated further. Of the three sites investigated, based on scans of the filters using PCM, no fibres were found on any of the filters collected from either Patumahoe (rural background) or Queen Street (urban roadside). Therefore, these filters were not subjected to further examination *via* the more time-consuming and expensive SEM-EDS method. By contrast, the Henderson (suburban roadside/light industry) filters did reveal fibrous materials across the surface. The morphology of the structures was found to be largely fibrous with some long, thin fibres mixed in with some hexagonal shards (Figures 6, 7, 9).

The clustered nature of the components in the image in Figure 7 suggests that the long fibre might have impacted first, and then other fibres and particles impacted on top during the 24-h sampling period. Within the image and EDS spectra from Figure 7, the fragment numbered “2” closely resembles that of the zeolite erionite, as exemplified in Figure 8. Although in its captured form it has the morphological dimensions of a particle, it was likely fibrous when entering the atmosphere given the fibrous nature of erionite in the bedrock (Patel et al., 2021). There are obviously limitations to identifying individual fibres with SEM/EDS alone, and the results should be seen as indicative only. However, these findings do suggest the need for further investigation.

Another air pollutant of growing concern is respirable silica particles. These often fibrous materials are long-lasting in the atmosphere and easily inhaled given their small dimensions (Gualtieri, 2018; Waeselmann et al., 2020). The methods used for this paper show that it is possible to confirm the presence of these potentially hazardous particles and adequately describe the morphological and chemical composition of silica fibres and fibre-like structures, thus helping to identify them on archived filters. Whether smaller fibres found in the ultrafine range would be visible is unclear, perhaps unlikely given the PTFE fibrous mesh structure. However, such fibres are generally rare with mineral fibres usually found to be in the larger (5–20 µm) size range.

Finally, the method of scanning filters first for fibre-like structures was instructive and useful as a first step. However, it cannot be discounted that some particles on the filter may actually be fibres that had been crushed or fragmented into smaller pieces. Such fragments would also be informative about the historical airborne loading of potentially hazardous fibrous forms.

## Conclusion

This paper presents the reanalysis of archived PTFE filters originally collected for the compositional analysis of Auckland’s ambient air particulate matter. Retrospective information on new air pollutants of interest, such as fibrous zeolites, was obtained, as demonstrated here by the identification on filters of previously unobserved fibrous materials.

The initial step of screening archived filters for analysis using those with the highest Si and Al was found to be somewhat successful. However, high soil content did not necessarily translate into fibres or fibre-like particles on all the chosen filters, with both mineral and non-mineral found in plentiful quantities within the historic Henderson samples, largely from the 2011 collection. A review of activities in the vicinity of the monitoring site during May 2011 will be undertaken with use of mapping, council records and press information (reporting fires, infrastructure construction, *etc.*).

The use of fibrous PTFE filters does increase the complexity of identifying fibres that are under a certain size, with fibres <1 µm being more likely to be embedded in the filter and, therefore, hard to distinguish from fibres from the filter itself. However, the use of PCM showed that the larger width of the fibre-like particles tended to allow them to sit on top rather than be embedded within the filter substrate. The thickness of the filters allowed only partial illumination from PCM, which could make the identification of smaller fibres difficult.

The subsequent use of SEM techniques for filters identified as interesting from PCM methods allowed for the identification of several fibrous mineral forms potentially including fragments of erionite and respirable silica. Given the limited number of fibres present, identification of the material or their origin was not possible with certainly. However, the presence of quarrying in the nearby vicinity, and the similarity of the EDS scan of zeolites within the filter to that of erionite found in Auckland’s bedrock, suggests that further investigation is warranted. These results open the door to the reanalysis of filters, collected initially for a different purpose, for emerging pollutants such as respirable silica, micro-plastics as well as mineral particles, to be identified using reasonably simple and quick methodologies. This study highlights the value in the retention and careful archiving of air quality filters for retrospective analysis for such a purpose.

## Data Availability

The original contributions presented in the study are included in the article/Supplementary Material, further inquiries can be directed to the corresponding author.
