# Supplementary material for: Reanalysis of historic elemental speciation filters to investigate the presence of fibrous mineral particles using microscopy techniques
Source: Front Chem. 2022 Nov 3;10:1032624. doi: 10.3389/fchem.2022.1032624 (PMC9669066; doi:10.3389/fchem.2022.1032624)
Supplement: Supplementary file 1 [file DataSheet1.docx]

**Supplementary Material**


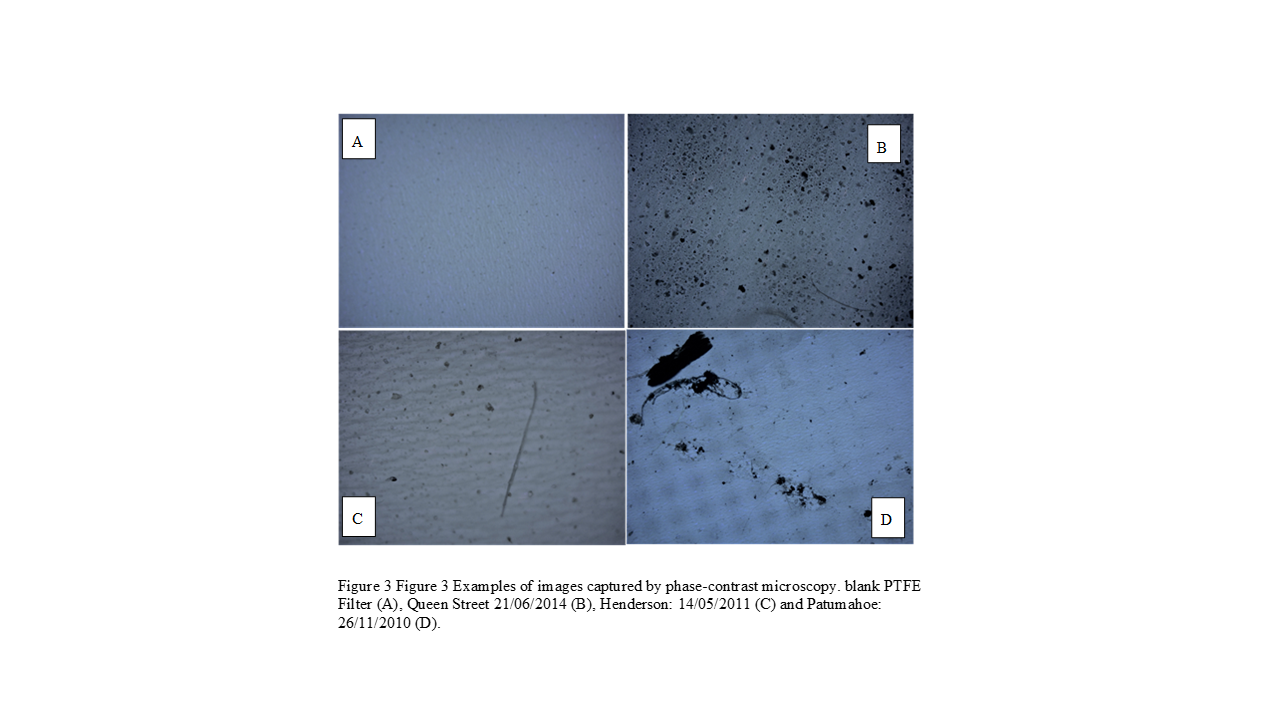


Figure S1 Examples of images captured by phase-contrast microscopy. From top left, blank PTFE Filter, Queen Street 21/06/2014 (top right), Henderson: 14/05/2011 (bottom left) and Patumahoe: 26/11/2010 (bottom right). Unfortunately, no calibrated scale bar was available at the time the images were taken.

Figure S2 Source contributions to PM10 at the Henderson, Queen Street and Patumahoe air quality monitoring sites.

Table S1 Filter samples with the ten highest Al, Si concentrations and crustal matter source contributions to PM10 at the Henderson, Queen Street and Patumahoe air quality monitoring sites

| **Henderson PM_10_ Al, Si and crustal matter source contribution concentrations** | | | |
| --- | --- | --- | --- |
| Sample date | Al | Si | Soil |
|  | ng m^-3^ | ng m^-3^ | µg m^-3^ |
| 02/12/2018 | 663 | 1659 | 9.3 |
| 09/01/2014 | 438 | 1051 | 6.5 |
| 03/10/2009 | 392 | 987 | 4.3 |
| 28/09/2006 | 338 | 982 | 4.1 |
| 08/05/2007 | 407 | 960 | 4.6 |
| 14/05/2011 | 321 | 816 | 3.3 |
| 24/07/2015 | 275 | 757 | 3.6 |
| 14/05/2013 | 227 | 750 | 2.9 |
| 05/02/2014 | 289 | 746 | 3.6 |
| 14/07/2009 | 248 | 743 | 3.0 |

| **Queen Street PM_10_ Al, Si and crustal matter source contribution concentrations** | | | | |
| --- | --- | --- | --- | --- |
| Sample date | Al | Si | Soil | Construction |
|  | ng m^-3^ | ng m^-3^ | µg m^-3^ | µg m^-3^ |
| 04/05/2007 | 1453 | 4827 | 17.4 | 12.9 |
| 02/05/2007 | 1305 | 4580 | 8.8 | 27.0 |
| 11/04/2007 | 992 | 3194 | 10.8 | 9.5 |
| 14/07/2014 | 324 | 2963 | 3.5 | 0.0 |
| 07/05/2007 | 1072 | 2886 | 12.2 | 6.7 |
| 30/08/2007 | 607 | 2733 | 0.9 | 24.8 |
| 28/09/2009 | 943 | 2374 | 11.8 | 0.0 |
| 29/08/2007 | 463 | 2090 | 1.0 | 18.1 |
| 23/08/2007 | 406 | 1942 | 3.5 | 9.5 |
| 01/09/2007 | 452 | 1807 | 0.3 | 18.6 |

| **Patumahoe PM_10_ Al, Si and source contribution concentrations** | | | |
| --- | --- | --- | --- |
| Sample date | Al | Si | Soil |
|  | ng m^-3^ | ng m^-3^ | µg m^-3^ |
| 09/03/2010 | 710.1 | 1354.2 | 20.9 |
| 08/03/2010 | 439.8 | 818.5 | 9.3 |
| 26/11/2010 | 397.5 | 725.0 | 10.8 |
| 19/03/2010 | 421.8 | 689.9 | 9.6 |
| 10/03/2010 | 368.4 | 684.7 | 9.9 |
| 25/11/2010 | 353.6 | 668.3 | 8.5 |
| 07/03/2010 | 375.5 | 657.1 | 9.3 |
| 11/12/2010 | 327.9 | 656.2 | 8.1 |
| 18/03/2010 | 378.2 | 651.5 | 10.7 |
| 04/01/2011 | 236.1 | 649.1 | 6.5 |

Table S2 Rudimentary fibre counts across the 47mm plane PTFE filters for the May 2011 filters from Henderson.

| **Date** | **Fibres detected?** | **Fibre count* (30 planes of vision)** | **Forward for SEM/EDS analysis** | **Notes** |
| --- | --- | --- | --- | --- |
| 02/052011 | Yes | 6 | Yes |  |
| 5/05/2011 |  |  |  | No Filter |
| 8/05/2011 |  |  |  | No Filter |
| 11/08/2011 | yes | 6 | yes |  |
| 14/05/2011 | yes | 32 | yes |  |
| 17/05/2011 |  |  |  | No Filter |
| 20/05/2011 | yes | 7 | Yes |  |
| 23/05/2011 | yes | 5 | Yes | a lot of BC on this filter further obscures light |
| 26/05/2011 | yes | 4 | Yes |  |
| 29/05/2011 | yes | 4 | no |  |
| *Note : Fibres are defined here as having a length to width ratio greater than 3:1 | | | | |
